# Levels and associated factors of the maternal healthcare continuum in Hadiya zone, Southern Ethiopia: A multilevel analysis

**DOI:** 10.1371/journal.pone.0275752

**Published:** 2022-10-10

**Authors:** Ritbano Ahmed, Mohammed Sultan, Selamu Abose, Biruk Assefa, Amanuel Nuramo, Abebe Alemu, Minychil Demelash, Shamill Eanga, Hassen Mosa

**Affiliations:** 1 Department of Midwifery, College of Medicine and Health Science, Wachemo University, Hossana, Ethiopia; 2 Department of Statistics, College of Natural and Computational Science, Wachemo University, Hosanna, Ethiopia; 3 Department of Anesthesia, College of Medicine and Health Sciences, Wolkite, Ethiopia; 4 Department of Midwifery, College of Medicine and Health Sciences, Werabe, Ethiopia; Ambo University, ETHIOPIA

## Abstract

**Background:**

The continuity of care throughout pregnancy, birth, and after delivery is an effective strategy to avert maternal and newborn deaths. A low proportion of mothers have achieved the continuum of maternal care in Ethiopia. This study aimed to assess the rate and factors associated with the completion of a continuum of maternal healthcare services in Hadiya Zone, Southern Ethiopia.

**Methods:**

A community-based, cross-sectional study was conducted over two months (from September to October 2021) in 18 kebeles of the Hadiya zone, southern Ethiopia. Multistage cluster sampling was carried out to select the required study subjects, and data were collected using a structured, interviewer-administered questionnaire. A multilevel binary logistic regression model was used to examine the effects of individual and community-level factors on key elements of the care continuum. The measure of fixed effects was expressed as an odds ratio with a 95% confidence interval (CI).

**Results:**

In this study, only 11.3% of women completed all components of the care continuum, which included four or more antenatal visits, skilled birth attendance, and postnatal care. The factors that are significantly associated with the completion of maternal care include higher maternal education [AOR = 4.1; 95%CI: 1.3–12.6], urban residence [AOR = 1.8; 95%CI: 1.1–3.0], time of first antenatal care follow-up [AOR = 2.7; 95% CI: 1.6–4.6], knowledgeability regarding postnatal danger signs [AOR = 1.9, 95% CI: 1.1–3.3], being in the highest wealth quintile [AOR = 2.8; 95%CI: 1.2–6.6] and primipara [AOR = 3.6; 95%CI: 1.4–9.4].

**Conclusion:**

The rate of continuum of maternal healthcare services utilization was low in the study area. The findings indicated that higher maternal education, urban residence, time of first antenatal care follow-up, knowledgeability regarding postnatal danger signs, being in the highest wealth quintile and primipara were the factors associated with the completion of the continuum of maternal care. As a result of this study’s findings, program planners and ministry of health and non-governmental organizations working on maternal health should prioritize continued and strengthened health education in order to increase the completion level of the continuum of maternal healthcare services.

## Introduction

Despite substantial improvements in global maternal and newborn health, millions of women and newborns die from preventable causes each year [1, 2]. In 2017, about 295,000 women died from pregnancy-related complications, and 94% of these deaths occurred in low- and middle-income countries (LMICs), with sub-Saharan Africa (SSA) accounting for nearly two-thirds of the deaths [1]. Nearly 25% of deaths occurred in the antepartum period, another quarter (25%) occurred in the intrapartum and immediate postpartum periods (up to 24 hours after delivery) and 33% occurred during the postpartum periods (24 hours to 42 days after delivery) [1]. About 2.5 million children died in the first month of life in 2018 [3], and 2.6 million babies were stillborn [4]. Neonatal deaths are heavily concentrated in LMICs; in SSA and South Asia alone, they accounted for 79% of neonatal deaths [3].

Ethiopia has significantly reduced its maternal mortality rate from the 1990s by an average annual rate of 70(5%) or more [1]. However, the levels of maternal mortality in Ethiopia remain among the highest in the world [5]. Evidence shows that the maternal death rates are high in Ethiopia during the antepartum, intrapartum, and postpartum periods, with death rates of 39.0%, 24.,3% and 43.2%, respectively [6]. In addition, Ethiopia, like most countries in SSA, is characterized by high neonatal deaths (30 deaths per 1,000 live births) [7], and worldwide, it recorded the fifth-highest number of newborn deaths in 2016 [8].

Based on the evidence to date, a continuum of maternal care approach could be used to address these risks and improve the health and survival of women, newborns and children worldwide. This is an innovation designed to support strategies to save the lives of women and their children [9, 10]. It is characterised by connections among levels of maternal care services (from pre-pregnancy to delivery and postnatal care) with access to different types of health services and activities at each level, including preventive, supportive and curative services at each level [9]. Evidence confirms that a reduction in maternal and neonatal deaths can be achieved by delivering high coverage with quality skilled care during pregnancy [1, 2, 11–14], childbirth[1, 2, 15, 16] and the postnatal period [1, 2, 7, 15]. Research has also demonstrated that providing continuity of maternal care reduces neonatal and perinatal mortality [17–19], preterm birth [20] and maternal mortality [17].

The government of Ethiopia has made maternal health a priority in its political agenda and has maintained its commitment to improving the health and survival of women and babies in the country. To reduce maternal mortality to 267 deaths per 100,000 live births, a set of high-impact interventions have been implemented, including antenatal care, skilled birth services and postnatal care [21]. Access to and utilisation of key health care services through Health Extension Workers, the government’s flagship programme, has improved. There has been an expansion of primary and secondary health care through the accelerated expansion of health centers and hospitals throughout the country. In addition, the country has equipped a large proportion of these facilities with basic equipment and supplies and has staffed them with a trained health workforce. Furthermore, health care finance has shown a large improvement, along with infrastructure and sanitation facilities. The health care delivery system has been augmented by private and non–governmental organisations [21, 22].

Even though the government has taken significant steps to increase the coverage of the continuum of maternal care, only a low proportion of mothers have achieved the continuum of maternal care in Ethiopia [23–26]. Research conducted using the 2016 EDHS shows only 9.1% of mothers complete all components of the continuum of maternal care [26]. In addition, several studies conducted to survey factors associated with maternal health service utilisation in Ethiopia and other developing countries have considered antenatal, delivery and postnatal care as separate entities at the design and/or the analysis stages [27–32]. Additionally, these previous studies focused mainly on individual-level factors, with little attention given to community factors and the service delivery environment. The evidence confirms that maternal health care services should be managed in a unified way to bring the intended interventions to mothers and newborns to improve their health and survival [11]. Moreover, many studies have used low-level analysis to identify contributing factors to maternal care service utilisation, but these studies did not appropriately handle hierarchical data. In this regard, an appropriate methodology is imperative for designing appropriate interventions to increase the coverage and contributing factors of the continuum of maternal care. Therefore, this study aimed to assess the level of the continuum of care and the effects of individual and community-level factors on the completion of the continuum of maternal healthcare services using multilevel logistic regression analysis.

## Methods and materials

### Study setting, design, period and population

A community-based cross-section study was carried out from September 1 to October 30, 2021, in Hadiya Zone, Southern Ethiopia. Its capital, Hossana, is 232 km away from Addis Ababa. According to the annual report of the Zone’s health department of 2013, Zone has 13 districts and four urban administrations and 329 kebeles (24 urban and 305 rural kebeles), with a total population of 1,767,390, of whom 49.56% are men and 50.44% women; 84.37% are rural inhabitants. In 2020/21, 56,697 (94%) of women had at least one ANC visit, 13,129 (18.8%) had ANC4+, and 50,355 (88%) were attended by a skilled birth attendant in a health facility (i.e., hospitals and health centres). The Zone has four hospitals, six centres and 311 health posts, which are all government-run. It also has one hospital, 63 private clinics and 44 pharmacies, all of which are private-own facilities [33].

The source population consisted of all women who had given birth in the Hadiya zone within 12 months before the study period and the study population consisted of randomly sampled women from this group. All women who had given birth in the Hadiya zone within 12 months before the study period were included in the study, and women with mental illness and those who were unable to hear and talk were excluded from the study because it was considered that they would not be able to provide the necessary information.

### Sample seize calculation and sampling technique

The sample size was computed using a single population proportion formula with the following assumptions: 9.7% proportion of completion of continuum of maternal care [23], 95% confidence interval (CI), 3% margin of error, 10% non-response rate and design effect of 2, the final sample size became 823.

Multi-stage sampling technique was employed. At the beginning, three districts were randomly selected from 13 districts (Lemo, Soro, and West Badwacho), along with Hossain, Gimbichu, Shone and Homecho from the town administration. Accordingly, from each district, three clusters (kebeles) and one center of the district (called a small town in this study) were selected. Similarly, six urban kebeles were randomly taken, three from Hossana and three from other town administrations. Consequently, a total of nine rural kebeles and nine urban kebeles (six from the big towns and another three from the small towns) were included in the study. All households with women who had given birth within 12 months before the data collection period in clusters (kebeles) were obtained from the respective kebele health post (family folders). Finally, a systematic sampling method was used to select women who had given birth within the last 12 months before the data collection period from all selected kebeles. If a household has more than one woman who has given birth within the last 12 months before the data collection, one of these women was selected using a simple random sampling method. Women who were not available in the household on two different visits (one week apart) were excluded from the study.

### Data collection procedure

A pre-tested face-to-face interviewer administered questionnaire was used to collect data, which was adapted from instruments used in related studies [23, 24, 34–38] as well as Ethiopian demographic and health survey data [5]. The questionnaire was primarily focused on individual and family factors, postpartum family planning, antenatal, delivery, and postpartum care and participant knowledge related to danger signs during pregnancy and childbirth. In this study, a total of 12 data collectors and five supervisors were involved (S1 File).

We used the following points to maintain data quality: the questionnaire was initially prepared in English, first translated into the local language, and then translated back into English by experts to check the consistency. The questionnaire was pretested on 5% of the sample size in the An-Lemo district, and necessary modifications were made for the local context before data collection. Additionally, the data collectors and supervisors were trained for a day by the investigators of this study on the content of the questionnaire and the manner of data collection. All of the completed questionnaires were checked daily for completeness, accuracy, clarity, and consistency by the supervisors and principal investigators. Furthermore, the completeness and consistency of the variables during data entry and analysis were confirmed using frequency distributions.

### Measurements

**ANC 4+**: ANC 4+ indicated mothers who had received four or more ANC visits in the previous pregnancy. Hence, this variable is coded as "1" if the respondent received four or more ANC visits. This variable is coded as "0" for mothers who have not received four or more ANC visits [23, 38]. **ANC 4+ to SBA**: AN 4+ to SBA referred to women who received continued care from pregnancy to facility delivery and had four or more antenatal care visits. Hence, this variable is coded as "1" if the respondent received at least four ANC follow-ups and a facility delivery, and "0" for receiving at least four antenatal care but not attending facility delivery (skilled birth attendance) [23, 38]. SBA to PNC within 48 hours after birth (CoC): Women were included who had received both at least four ANC visits and facility-based delivery services to identify factors associated with women’s retention in PNC visits within 48 hours after birth. The two categories of the outcome are "1" for receiving four or more antenatal care, skilled birth attendance, and postnatal care within 48 hours (completing the continuum of maternal care), and "0" for receiving at least four antenatal visits and skilled birth attendance but not postnatal care within 48 hours after birth [23, 24, 37, 39].

**Knowledge on pregnancy danger signs**: In this study, danger signs during pregnancy were assessed using 7 items, with a correct answer given a score of "1", and an incorrect answer given a score of "0". A woman was classified as knowledgeable if she spontaneously mentioned at least five of the seven key danger signs of pregnancy; if not she was classified as not knowledgeable [23]. **Birth preparedness and complication redness**: A woman was considered "well prepared’ for birth and its complications when she reported that she had implemented five or more components of birth preparedness and complication readiness; otherwise she was considered "not well prepared [23]. **Knowledge on postpartum danger signs**: In this study, danger signs postpartum were assessed using 5 items, with a correct answer given a score of "1", and an incorrect answer given a score of "0". A woman was categorized as knowledgeable to obstetrics danger signs of postpartum if she could answers correctly a minimum of three of the questions she asked [23, 24].

#### Household wealth index

Using principal component analysis, the study women’s household wealth status was created (PCA). A total of 26 factors, including household utilities, productive and non-productive assets and miscellaneous items, were first utilized to assess household wealth status. The factors that can discriminate between relatively "rich" and relatively "poor" families are then chosen using descriptive analysis. The analysis did not include any variables or assets that were owned by more than 95% or less than 5% of the sample. The Kaiser-Meyer-Olkin (KMO) measure of sampling adequacy (0.6) was used to determine whether PCA assumptions were satisfied, and our value of 0.79 is satisfactory. The variables were eliminated from the correlation matrix when the correlations were too strong (above 0.9) or excessively low (below 0.1). The wealth index, which reflects the household’s wealth, is based on the first primary component because it accounts for the biggest share of the overall variance. The first of them contributed the most to the variance’s explanation, at 34.6%. Farmland ownership, mobile and cell phone ownership, and radio and television ownership were the only things to remain in the first component. This component’s factor score was used to categorize the study women’s household wealth into quintiles. **Distance from health facilities**: In this study, the respondents were asked about how long it took them to walk to the closest health facility in order to assess the distance. The people who claim it takes less than or equal to 30 minutes are then categorized as being close, and the people who claim it takes more than 30 minutes are categorized as being far [34].

### Statistical analysis

The EpiData version 3.1 software was used for data entry, and the SPSS version 24.0 was used for data analysis. Descriptive analysis was performed to describe the levels of key maternal and reproductive services and the continuum of maternal care. Women living in the same kebele may share similar characteristics; hence, the estimates from ordinal regression that assume all individuals are independent would not be efficient. Because women are nested within households and households are nested within kebeles, multilevel binary logistic modelling appropriately take the hierarchical structure of the data into consideration. This model also enables partitioning of the total variation in the outcome into within-group (i.e. kebele) and between-group components, which allows for differentiating the relative contributions of level 1 and level 2 variables.

At the beginning, the null model (excluding all explanatory variables) was fitted to determine if the grouping variable at level 2 significantly affects the intercept of the dependent variables (to make a decision whether multilevel modeling is needed or not). The result of the null/empty model indicated the variance component for the grouping variable (kebele) is significant in the random effects table (p < 0.05), then there is an effect of the higher level on the outcome variables at a lower level and therefore multilevel modeling is necessary. Then, model II (i.e., the full model/random intercept) was included, including both level 1 and level 2 variables in addition to kebele-specific random effects for all the three outcome variables. A chi-square test was also used to select candidates for multilevel binary logistic regression based on unadjusted relationships between each outcome variable and predictor factors. Then variables with P–values of 0.25 or less were entered into multilevel binary logistic regressions to assess the association after adjusting for other variables. The fixed effects were reported in terms of odds ratios with their P-values and 95% CIs, while the interclass correlation coefficient (ICC) was used to compare the proportion of variance caused by the random intercept at the kebele level among different models.

### Ethics approval and consent to participation

Ethical approval was granted by the Research Ethical Review committee of Wachemo University. Moreover, permission letters were obtained from the Hadiya Zonal Health Department, each health office, and selected kebele administration before data collection began. Finally, written informed consent was obtained from each participant included in the study after its objectives were explained to them. Participants were accordingly informed about the purposes, procedures, potential risks, and benefits of the study. Confidentiality was maintained throughout the study by excluding personal identifiers, such as names and addresses.

## Results

### Socio-demographic characteristics

Among 823 selected participants, seven mothers (0.85%) refused to be interviewed. The mean age of mothers was reported as 28.9 years ±4.3 (standard deviation), with 373 (45.7%) participants falling within the age range of 25–29 years. Of the participants, a majority of 811 (99.4%) were married, 683 (83.7%) were of Hadiya ethnicity, 695 (85.2%) were Protestants (in terms of their religion) and 611 (74.9%) were housewives. In terms of education, 10.7% had completed higher education, 10.9% had a secondary or preparatory level of education and 47.1% had a primary level of education, while 15.7% were able to read and write. There were 477 (58.5%) participants who resided in rural areas and 14.7% were in the highest wealth quintile. In terms of maternal and newborn health care accessibility, more than half of the women (56.4%) stated that it took less than 30 minutes to travel to a health care facility and 54.5% said they discussed maternity care with their husbands. Other sociodemographic characteristics are shown in Table 1.

**Table 1:** Socio-demographic characteristics of mothers who have given birth in the last 12 months in the Hadiya zone, 2021.

Characteristics		Frequency(n = 816)	Percent (%)
Age in year	15–24	104	12.7
	25–29	373	45.7
	30–34	230	28.2
	> = 35	109	13.4
Religion	Protestant	695	85.2
	Orthodox	50	6.1
	Muslim	56	6.8
	Catholic	15	1.8
Ethnicity	Hadiya	683	83.7
	Kambata	30	3.7
	Amhara	32	3.9
	Site	40	4.9
	Gurage	14	1.7
	Others	17	2.0
Educational status of husbands	Cannot read and write	61	7.5
	Read and write	112	13.7
	Primary education	364	44.6
	Secondary education	180	22.1
	Higher education	99	12.1
**Mothers’ Occupation**	Housewife	611	74.9
	Merchant	102	12.5
	Government Employee	61	7.5
	Daily laborer	42	5.1
Husband’s occupation	Farmer	508	62.3
	Merchant	90	11.0
	Employee	118	14.5
	Daily laborer	100	12.3
Wealth quintile	Lowest	187	22.9
	Second	154	18.9
	Middle	185	22.7
	Fourth	170	20.8
	Highest	120	14.7
Duration takes to reach nearest health facilities	<30 minutes	460	56.4
	> = 30 minutes	356	43.6
Exposure to mass media**	Yes	418	51.2
	No	398	48.8
Mode of transportation to health facilities	on foot	455	55.8
	Motor/ /Bajaj/car	361	44.2
Information regarding CBHI	Yes	548	67.2
	No	268	58.6
CBHI status(n = 548)	Insured	321	41.4
	Not insured	227	27.8

**Refers to reading a newspaper or watching TV or listening to the radio at least once a week.

### Obstetric characteristics

Of the 816 study participants, 267 (32.7%) were primigravida and 142 (17.4%) were grand multipara. Five hundred and ten (62.5%) participants had a history of using family planning methods before their previous pregnancy. Considering the knowledge level of the participants, 55.6% and 44.4% of the women had high and low levels of knowledge toward dangers signs in pregnancy, respectively (Table 2).

**Table 2:** Obstetrics and maternal health care service characteristics of mothers who have given birth in the last 12 months in the Hadiya zone, 2021.

Variables		Frequency	Percent (%)
Previous use of the modern contraceptive method	Yes	510	62.5
	No	306	37.5
Parity	Primipara	267	32.7
	Multipara	407	49.9
	Grand-multipara	142	17.2
Pregnancy status	Wanted	603	73.9
	Unwanted	213	26.1
ANC visits	No	54	6.6
	Yes	762	93.4
Time for first ANC visit	<16 weeks of GA	177	23.2
	≥16 weeks of GA	585	76.8
Birth preparedness and complication readiness	Not prepared	361	44.2
	Well prepared	455	55.8
Knowledge of obstetric danger signs of pregnancy period	Not knowledgeable	454	55.6
	Knowledgeable	362	44.4
Place of last delivery(SBA)	Home	296	33.3
	Health facilities	520	63.7
Knowledge of obstetric danger signs of the post-partum period	Not knowledgeable	487	59.7
	Knowledgeable	329	40.3
Postnatal care	No	645	79
	Yes	171	21.0
First PNC visit(n = 171)	Within two days, 3	28	16.4
	Three–seven days,	35	20.5
	Seven days to two weeks	31	18.1
	Two–six weeks postpartum	77	45.0
Decision-maker regarding MNC at home	Husband only	271	33.2
	A woman alone	100	12.3
	Both	445	54.5

### Overall use of maternity health care services in the study area

#### Antenatal care (ANC) follow-up

A significant proportion of the women, 762 in total (78.4%), had attended an antenatal care follow-up appointment, of which 426 (55.9%) attended a health centre and 336 (44.1%) attended a hospital. Of the women who had attended an ANC follow-up appointment, only 177 (23.2%) mothers had their first ANC visit before 16 weeks of gestation and 584 (76.8%) had it at, or after, 16 weeks of gestation (Table 2). Women who received all key ANC services amounted to 369 (48.5%). With respect to the specific services provided during ANC visits, 84.5% of mothers had their blood pressure taken, 88.3% had blood samples taken, 83.3% were vaccinated against tetanus toxoid (TT2+), 78.1% were tested for HIV, 68.8% were given 90+ iron, 66.4% received health information about danger signs and nutrition during pregnancy and 52.2% underwent urine analysis.

#### Skilled birth attendance

According to this study, 520 (63.7%) women gave birth to their previous child at a healthcare institution, with 79.0% of deliveries staff members such as nurses and health officers (16.2%). Among those women who were attended to being spontaneous vaginal births, 14.6% being instrumental vaginal births and the remaining 6.3% being caesarean births. Half of them (50%) gave birth at government health centres, 48.2% at government hospitals and the remaining 1.8% at private healthcare institutions. The women who gave birth at health facilities were attended by a midwife (52.4%), a doctor (31.4%) or or other by a skilled provider at a healthcare facility, 99.4% were attended to according to the plan and without any problems during labour. Ninety three (11.4%) women faced obstetric complications during the current pregnancy or labour, with 24 (25.8%), 21 (25.6%), 19 (20.4%), 15 (16.1%) and 14 (15.1%) women encountering hypertensive disorders, antepartum haemorrhage, premature rupture of fetal membranes, malpresentation and prolonged labour, respectively.

#### Postnatal care utilisation

Overall, 171 (21.0%) women received postnatal care, with 85% receiving their first PNC within the initial 48 hours after birth. Of the women who had at least one postnatal visit, 16.4%, 20.5%, 18.1% and 45.0% had their first PNC contact within two days, 3–7 days, seven days to two weeks and 2–6 weeks postpartum, respectively (Table 2).

### Continuum of maternal care

In this study, 336 (42.1%) mothers had four or more ANC, 30.6% gave birth with skilled health personnel in attendance, while 11.5% left the programme early. Another 19.3% did not continue to receive PNC after delivery. Only 11.3% of women completed the entire maternity care continuum (S1 Fig). According to the findings, 6.0% of mothers did not receive any maternal healthcare services across entire care, while 8.2% received only ANC4+ but not SBA or PNC. The percentages of women who received various combinations of maternity services are shown in Table 3.

**Table 3:** Percent distribution of women by different types of maternal health services received for the most recent birth, Hadiya zone 2021.

ANC1	ANC4+	SBA	PNC	%
-	-	-	-	6.0
**+**	-	-	-	17.9
**+**	**+**	-	-	8.2
**+**	-	**+**	-	26.7
**+**	-	-	**+**	1.8
-	-	-	**+**	0
-	-	**+**	-	0.24
**+**	**+**	**+**	-	19.4
-	-	**+**	**+**	.4
**+**	-	**+**	**+**	5.6
**+**	**+**	-	**+**	1.8
**+**	**+**	**+**	**+**	11.3
Total				100%
Total number of women				816

-Did not receive the service, + received the services

### Regression analysis results

As described in the procedure section, three random logit regression models were fitted to identify the characteristics that help or prevent women from receiving maternal healthcare services. To evaluate whether multilevel modelling was required, a null model was first fitted to each outcome variable. The null model revealed that there was a significant degree of variation for ANC4+, SBA and PNC across clusters (P < 0.05), indicating that a multilevel model should be developed. Therefore, a multilevel binary logistic model was fitted for all the outcome variables. In the null model, the ICCs for ANC4+, retention of SBA and PNC were 0.224, 0.227 and 0.273, respectively, indicating that between-kebele variation contributes to 22.4%, 22.7% and 27.3% of the total variation in ANC4+ uptake, retention of SBA and PNC, respectively. The results of the null/empty model are presented in Table 4.

**Table 4:** A multilevel model of retention in the continuum of maternal healthcare services pathway (ANC, SBA, and PNC): An empty model without covariates in the Hadiya zone, 2021.

Random effect parameter	ANC4+	Retention on SBA	Complete COC
Lvel-2 variance (95%CI)	0.95*(0.42, 2.16)	0.97*(0.42, 2.23)	1.21*(0.45, 3.28)
Rho-ICC	0.224	0.227	0.273
−2LL	3652.3	3764.8	4346.2

CI = Confidence interval, 2LL Log-likelihood,

*p < 0.05;

ICC (p) = Intra-class correlation Coefficient

Model I was used to determine the factors that influence the use of ANC4+. Individual-level characteristics linked to the use of ANC4+ included maternal age (25–29 and 30–34 years), maternal education (higher maternal education), status of pregnancy (planned pregnancy), parity (primipara and multipara) and time of the initial ANC check–up (before 16 weeks of pregnancy). Urban residences and proximity to healthcare facilities were the two community-level characteristics linked to the usage of ANC4+. The Rho-ICC of this model was 0.215, indicating that the variance between kebeles accounted for 21.5% of the entire variation in ANC4+ usage. Women aged 25–29 years and 30–34 years [AOR = 2.0; 95% CI: 1.1–3.6] and [AOR = 2.0; 95% CI: 1.1–3.7], respectively, were twice as likely to utilize ANC4+ compared to women aged ≥35 years. ANC4+ uptake was three times as frequent in mothers who had completed higher education [AOR = 3.1; 95%CI: 1.2–8.0] than among mothers who could not read or write. Primipara and multipara mothers were more likely to employ ANC4+ [AOR = 3.5; 95% CI: 1.8, 6.6] and [AOR = 2.8; 95% CI: 1.5–5.5], respectively than grand multipara mothers. Women with planned pregnancies were more likely to use ANC4+ [AOR = 3.2; 95% CI: 2.0–5.2]. Furthermore, mothers who began their first ANC visit before 16 weeks of pregnancy were nearly four times more likely to use ANC4+ than their counterparts [AOR = 3.5, 95% CI: 2.2–5.6]. This study also found that mothers who lived in urban areas were more likely to use ANC4+ than their counterparts [AOR = 2.0; 95% CI: 1.3–3.3].

Model II analysed the factors associated with the continuation of care from pregnancy to skilled birth attendance among women who had received at least four or more ANC visits. In the adjusted full multilevel binary logistic model, individual-level factors significantly associated with the retention of care from ANC to an institutional delivery were maternal age (30–34 year), parity (primipara and multipara), presence of obstetrical complications during pregnancy, the timing of the first ANC visit (before 16 weeks of gestation) and nature of pregnancy(planned). The community-level factors associated with the retention of care from ANC to an institutional delivery were urban residences and proximity to healthcare facilities. In this model, the Rho-ICC was 0.184, indicating that the variation between kebeles accounted for 18.4% of the total variation in the continuation of care from ANC4+ to skilled birth attendance. Planned pregnancies, primipara, multipara, maternal age of 30–34 years, having the first ANC visit before 16 weeks of gestation and the presence of obstetric complications during the current pregnancy were increasingly found to be the individual-level factors promoting the retention of care from ANC to an institutional delivery [AOR = 2.0, 95% CI: 1.2, 3.3], [AOR = 4.5; 95% CI: 2.3–9.0], [AOR = 2.4; 95% CI: 1.3–4.7], [AOR = 2.0, 95%CI:1.1, 3.7]. Among the community-level variables, living in urban areas and living close to healthcare facilities [AOR = 3.0; 95% CI: 1.8–4.7] and [AOR = 3.4; 95% CI = 2.5–5.7], respectively were factors contributing to the retention of care from ANC4+ to SBA.

Model III analyzed the factors associated with the continuation of care from delivery to the post-delivery period among women who received both ANC4+ and skilled birth attendance. Maternal education, parity, household wealth index, knowledge of danger signs in the postpartum period, and timing of the first ANC were individual-level factors associated with the completion of maternal care along a continuum of care. Living in urban areas and being close to health care facilities were community-level factors associated with completing maternal care along a continuum of care. In this model, the Rho-ICC is 0.24, indicating that there are significant differences between kebeles, with nearly a quarter (24%) of the variation in the use of PNC among mothers who got both ANC4+ and skilled birth attendance occurring between kebeles. Despite this, we can see that after correcting for level-1 and level-2 predictors, the reduction from the null model (ICC was 0.273). Higher maternal education was significantly associated with higher odds of completion of a continuum of maternal care services [AOR = 4.1, 95% CI, 1.3–12.6]. Primipara was 3.6 times more likely to complete the continuum of maternal health care services [AOR = 3.6, 95% CI: 1.4–9.4]. Similarly, the prevalence of a higher household wealth index also increased the likelihood of completion of a continuum of maternal care services [AOR = 2.8, 95% CI: 1.2–6.6]. Moreover, the likelihood of completing a continuum of maternal care services increased in mothers whose knowledge score of danger signs in the postpartum period was higher [AOR = 1.9, 95% CI: 1.1–3.3] and who had started ANCs prior to the 16^th^ week of gestation [AOR = 2.7, 95% CI: 1.6–4.6]. Mothers living in urban areas were 80% more likely to complete such a continuum of maternal care services [AOR = 1.8, 95% CI = 1.1–3.0]. Furthermore, the likelihood of completing a continuum of maternal care services increased in mothers who had lived close to health facilities [AOR = 2.3, 95% CI: 1.2–4.7] (Table 5).

**Table 5:** Results of multilevel binary logistic regression analysis on the continuum of the maternal care pathway among participants in the Hadiya zone, 2021.

Independent variables	Model I	Model II	Model II
	ANC4+	ANC4+ & SBA	ANC4+ & SBA & PNC(CoC)
	AOR(95%CI)	AOR(95%CI)	AOR(95%CI)
**Fixed effect**			
**Individual factors**			
**Mother’s age(ref = ≥35**)			
15–24	1.5(.7, 4.0)	0.8(0.3, 2.6)	0.6(0.3, 1.9)
25–29	2.0(1.1, 3.6)*	1.3(0.7,2.6)	0.6(0.3, 1.4)
30–34	2.0(1.1, 3.7)*	2.1(1.1, 4.2)*	0.8(0.3,1.8)
**Mother’s education(ref = Cannot read and write)**			
ad and write	1.3(.6, 2.6)	0.8(.4, 1.6) .8	1.1(0.4, 3.2)
Primary	.9(.5, 1.6)	0.8(.4, 1.2)	1.5(0.7, 3.4)
Secondary	.7(0..3,1.6)	0.5(.2, 1.1)	.9(0.3, 3.1)
Higher	3.1(1.2, 8.0)*	1.4(.3, 1.9)	**5.1(2.0, 13.3)** **
**Mother’s occupation(ref. = Employed)**			
Housewife	.7(.2, 2.9)	.5(0.1, 1.9)	0.4(0.5,2.6)
Merchant	0.4(0.1, 1.3)	0.4(0.1, 1.1)	1.9(0.7, 1.9)
Daily laborer	1.1(.3, 3.1)	0.6(0.2, 1.6)	0.3(0.2,3.3)
**Husband’s occupation(ref. = farmer**)			
Merchant	0.7(0.3,1.5)	0.8(0.4,1.7)	0.2(0.1,3.7)
Employed	0.8(0.3, 1.3)	0.9(0.4, 1.9)	1.5(0.5, 3.2)
Daily laborer	0.3(0.1, 1.6)	0.5(0.2,1.3)	1.5(0.4,4.5)
**The final decision on MCH(ref. = husband)**			
Woman	1.0(.5, 1.9)	.5(0.3,1.8)	.5(0.2, 1.2)
Both	.6(.4, 1.9)	1.0(.5, 1.9)	1.4(0.6, 3.4)
**Wealth quintile(ref = Lowest)**			
Second	0.9(0.5, 1.6)	1.1(0.6,2.0)	1.8(0.8, 4.2)
Middle	1.1(0.6, 1.9)	1.1(0.7, 2.3)	1.6(0.7, 3.6)
Fourth	1.1(0.6,2.1)	1.3(0.7,2.3)	1.4(0.6, 3.2)
Highest	1.2(0.6, 2.3)	1.1(0.6,2.1)	2.8(1.2, 6.6)*
**Parity (ref. = grand multipara)**			
Primipara	3.5(1.8, 6.6)**	4.5(2.3, 9.0)**	3.6(1.4, 9.4)**
Multipara	2.8(1.5, 5.1)**	2.4(1.3,4.7)**	2.4(0.9, 6.3)
**Pregnancy status(ref. = Unwanted**			
Wanted	3.2(2.0, 5.2)**	2.0(1.2, 3.3)*	1.3(0.6, 2.6)
**Timing of first ANC(ref. = At or more than 16 weeks)**			
Less than 16 weeks	3.5(2.2, 5.6)**	4.6(2.9, 7.2)**	2.7(1.6, 4.6)**
**Complications encountered during the last pregnancy(ref. = No)**			
Yes	1.2, (.6, 2.3)	2.0(1.1, 3.7)*	0.9(0.5, 1.8)
Knowledge of danger signs of postpartum(ref. = Not Knowledgeable)			
Knowledgeable	__	__	1.9(1.1,3.3)*
**BPCR(ref. = not prepared)**			
Prepared	__	1.3(0.8, 2.0)	0.8(0.5, 1.3)
**Community-level factors**			
**Residence (ref = Rural)**			
**Urban**	2.0(1.3, 3.2)**	3.0(1.8, 4.7)**	1.8(1.1, 3.0)*
**Distance from health facilities(ref.≥ 30 minutes)**			
<30 minutes	5.3(3.3, 8.5)**	3.4(2.1,5.7)**	2.3(1.2, 4.7)*
**Random effects**			
Variance(τ^2^0)	.9(.37,2.16)	0.77(0.31,1.93)	1.03(0.35,3.0)
Rho-ICC	0.215	0.184	0.24
-2LL	4141.9	4198.5	4596.0

***p<0.01,

**p < 0.05,

ICC: Intra correlation Coefficient;-2LL: -Log likelihood

## Discussion

The finding of this study showed that only 11.3% of women completed all the stages of the requisite continuum of maternal healthcare services. The completion of all components of maternal care in the present study is relatively similar to that reported in studies conducted in Ethiopia (9.1%) [26], Gondar (12.1%) [23], Arba-Minch (9.7%) [24], Ghana (7.9%) [40], Tanzania (10.3) [41] and Nigeria (11.4%) [42].

This finding was lower than the study carried out in Dabat district (21.6%) [34], Debre Birhan (37.2%) [35], Wayu district (Ethiopia) (16.1%) [36], Nepal (24.7%) [37], Cambodia (60%) [38] and Pakistan (27%) [43]. This lower percentage might be due to socio-economic factors or effort given in the completion of all components of maternity care by the concerned body, especially regarding postnatal care or cultural differences, as some previously conducted studies were conducted in middle- and high-income countries, or to various interventions carried out in these study areas. The other studies additionally incorporated women who had received at least one ANC in the continuum of care cycle, done purely in urban areas and women who received postnatal care within the first six postnatal weeks, but we included only women who received ANC4+ to PNC within the first 48 hours.

In contrast, this study found a higher rate of completion of the continuum of maternal healthcare services compared to other studies in Cambodia and Korea, which were 5.0% [39] and 6.8% [44] respectively. Variations in the research setting, time, and sample size may help to explain this discrepancy, and it’s also possible that sociodemographic differences are responsible.

Residing in an urban area was significantly associated with the completion of continuum of maternal healthcare services. This finding is supported by studies conducted in Gondar and Wayu distric, Ethiopia [23, 36], Nepal [37], Cambodia [38] and Nigeria [42], which all reported that urban residences are more likely to receive all of the components of a continuum of maternal health care services. One possible explanation for women’s retention throughout the continuum of maternal care services is that urban women are more knowledgeable about maternal health services during pregnancy, delivery, and postpartum care and, as a result, use complete care maternal health care services more frequently. Another reason may be that urban women are more likely to receive information regarding maternal health care services, which results in increased utilisation of all components of the continuum of care.

Women within the age groups 24–29 and 30–34 were more likely to receive ANC4+ than women aged 35 or older, a finding that was similar to those of studies in Gondar, Cambodia, Tanzania, Nigeria and Pakistan [23, 38, 41–43]. A possible reason for this finding is that younger women have less experience giving birth and may have more information about all three components of maternal health care services along their pathway of care, while older women may have more experience giving birth, which may decrease utilisation. Another possible explanation for this is that, as age increases, a woman’s confidence and experience may increase; as a result, the probability of using maternal health care services reduces.

Obstetric complications during pregnancy were significantly associated with facility delivery retention. This finding is similar to that of the studies conducted in Ethiopia [23] and Tanzania [41]. A probable explanation is that women who experienced obstetric complications expect an increased level of care; therefore, they are retained in the continuum of care.

The uptake of ANC4+ and continued facility delivery were found to be associated with planned pregnancy and living close to health facilities. Similarly, a study conducted in Gondar, Ethiopia [23] found that planned pregnancy was significantly associated with both the use of ANC4+ and continued facility delivery. The possible reason for this finding might be because women who plan to have a child might want to have a healthy pregnancy and child and thus, they might pay great attention to maternal health care services. Women who are living far from a health facility may be discouraged from going to a health facility due to their limited access to health care services.

Primipara was more likely to complete continuum of maternal healthcare services. This finding is parallel to those of studies conducted in Pakistan [43] and Cambodia [38]. The possible explanation for this is that, as parity increases, a woman’s confidence and experience may increase. As a result, the probability of receiving maternal health care services decreases for women who have given birth four or more times.

Women who had their first ANC visit before 16 weeks of gestation were reported to have a higher ratio of completion of continuum of maternal healthcare services. This finding is also supported by studies conducted in Gondar, Arba-Minch and Zambia, which reveal that those women who started their first ANC visit before 16 weeks of gestation were more likely to accomplish all three components of care than those who started their first ANC after 16 weeks [23, 25, 44]. This might be due to the early initiation of ANC visits, which allows the women to be counselled on the components of maternal health care services and the benefits of receiving all components of care. In addition, mothers who had ANC follow-up were financially and emotionally prepared for the demands.

The women who had a high level of knowledge about postpartum danger signs were more likely to complete continuum of maternal healthcare services compared with non–knowledgeable women. This is comparable with studies done in Gondar, Ethiopia [23] and Cambodia [38, 39]. This might be due to the fact that the more the mother knows about the danger signs, the more they use the care. In addition, women who are knowledgeable about danger signs are more likely to work on the prevention of danger signs before their occurrence.

The highest household wealth status was associated with the completion of the continuum of maternal healthcare services. This finding is again supported by studies conducted in Gondar, Pakistan, Nepal and South Asia and Sub-Saharan Africa, revealing that those women who had the highest household wealth quintile were more likely to use the continuum of maternal care [23, 37, 40, 45]. This finding is well known in the literature. This might be due to the fact that women with higher income can pay for health services and transportation.

Higher educated women had a better continuum of maternal health service utilisation compared with non-educated women. Women with more education are more likely to learn about health and health risks, to have more accurate health beliefs and knowledge and to be able to understand health care issues. This finding was found to be an almost universal fact and has been revealed in many studies [23, 37, 40, 44, 45].

### Limitations of the study

Since this study was conducted on mothers who had given birth up to 12 months before data collection, there could be recall biases in reporting their pregnancy and postnatal experiences. Furthermore, the social desirability bias might be a problem. Also, this study was not supported by a qualitative method; therefore, it was not possible to demonstrate detailed reasons and different perspectives for not completing the continuum of maternal care. Finally, only two community factors were studied in the research, another limitation.

## Conclusion

The rate of continuum of maternal healthcare services utilization was low in the study area. The findings indicated that higher maternal education, urban residence, time of first antenatal care follow-up, knowledgeability regarding postnatal danger signs, being in the highest wealth quintile and primipara were the factors associated with the completion of the continuum of maternal healthcare services. As a result of this study’s findings, program planners and ministry of health and non-governmental organizations working on maternal health should prioritize continued and strengthened health education in order to increase the completion level of the continuum of maternal care. Health institutions place emphasis on increasing the timely initiation of first antenatal care follow-up using different methods of information dissemination, counseling, and health education. The findings of this study also indicate how the targeting of more rurally located women who have been living far from suitable health facilities may improve retention in the continuum of maternal health care pathways. Finally, we recommend further researches to be done, including health facility factors and areas supplemented with qualitative data.

## Supporting information

S1 File Consent form, English and Amharic questionnaire. (DOCX)

S2 File SPSS. (SAV)

S1 Fig Level of retention on the continuum of the maternal care pathway among participants in the Hadiya zone, 2021. (DOCX)

## Abbreviations

ANC: Antenatal care
AOR: Adjusted odds ratio
CI: Confidence interval
EDHS: Ethiopia demographic and health survey
ICC: Interclass correlation coefficient
LMICs: Low- and middle-income countries
OR: Odds ratios
PNC: Postnatal care
SBA: Skilled birth attendance
SNNPR: Southern Nations, Nationalities, and Peoples’ Region
SPSS: statistical package for social sciences
SPSS: Statistical package for social sciences
SSA: Sub-Saharan Africa
