# Supplementary material for: Levels and associated factors of the maternal healthcare continuum in Hadiya zone, Southern Ethiopia: A multilevel analysis
Source: PLoS One. 2022 Oct 10;17(10):e0275752. doi: 10.1371/journal.pone.0275752 (PMC9550044; doi:10.1371/journal.pone.0275752)
Supplement: S1 File — (DOCX) [file pone.0275752.s001.docx]

# **Annexes**

**Annex I. English questionnaire and consent form**

**Dear Respondent:**

My name is _____________________This questionnaire is prepared to assess the women’s retention on the continuum of the maternal care pathway in Hadiya Zone, Southern Ethiopia. You are selected and included in the study as part of the sample population to complete the questionnaire designed by the researcher(s).

**Study title:** Women’s Retention on the Continuum of the Maternal Care Pathway in Hadiya Zone, Southern Ethiopia: A Multilevel Analysis

**Purpose:** This study will assess the level of women’s service utilisation and the effects of individual and kebele-level (i.e. cluster-level) factors on using key elements of the continuum of maternal care. The information obtained in this study will be used only for research purposes.

**Procedures**: There are questions that assess women’s retention on the continuum of the maternal care pathway in Hadiya Zone. I would like to ask you to give your genuine and honest answers on the questions forwarded. Because the data you will provide is very helpful to achieve the intended objectives of the study. If you need clarification please ask me. It will take you about 30 minutes to finish this survey.

**Benefits and Risks**: By participating in this study and answering our questions, you will not receive any direct benefit. However, the information will help the researcher to understand associated factors with women’s retention on the continuum of the maternal care pathway in order to appropriately identify future interventions related to problem to be found. Your participation in this study will not involve any risks. If a question makes you feel uncomfortable, you ma y choose not to answers.

**Confidentiality**: You will not be asked your name on to be written the survey questions. Any information obtained will be kept strictly confidential and will not be exposed to any other body. **Participation**: Involvement in this study is optional and in voluntary basis and you can drop any individual question or the whole questionnaire. But your participation and contribution in the study is very important to come up with important findings which may help local health planners to intervene the problem locally. If you would like to know more, please contact:

**Address of the Principal Investigator**: Ritbano Ahmed (Assistant professor), Mobile: +251910143410.

I thank you in advance for taking your time to answer questions.

Do you have any opinion regarding this study?

Do you agree to participate in this study?

Yes, Signature_______________ No, thank you!

Name of the data collector_____________________________ Sign________ Date______

**Instruction: Circle the appropriate answer** provided and where applicable writes the required responses in the spaces provided.

| Part I Socio-demographic Characteristics of the respondents | | | |
| --- | --- | --- | --- |
| S.N | **Questions and filters** | **Response and Code** | **Skip** |
| 100 | Place of residence | 1. Urban area  2. Rural area |  |
| 101 | Age of the mother in years | 1._________ Years  **OR**  99. I do not know |  |
| 102 | What is your current marital status? | 1. Single//Never married 2. Married 3. Divorce 4. Widowed |  |
| 104 | What is you religion? | 1. Protestant 2. Orthodox 3. Muslim 4. Catholic 5. Others (specify)----------- |  |
| 105 | What is your ethnicity? | 1. Hadiya 2. Kambata 3. Amhara 4. Silte 5. Others (specify)_______________ |  |
| 106 | What is the highest GRADE you completed? | 1. Cannot read and write 2. Read and write 3. Primary education 4. Secondary education 5. Higher education |  |
| 107 | What is your husband’s educational status? | 1. Cannot read and write 2. Read and write 3. Primary education 4. Secondary education 5. Higher education |  |
| 108 | What is your occupation, that is, what kind of work do you mainly do? | 1. Housewife 2. Farmer 3. Merchant 4. Student 5. Government Employee 6. Daily laborer 7. Other (specify)---- |  |
| 109 | What is your husband’s occupation? | 1. Farmer 2. Merchant 3. Student 4. Government Employee 5. Daily laborer 6. Other (specify)---- |  |
| 110 | How many family members are there in your house hold? | _______ numbers |  |
| 112 | Who were decision maker for the health care seeking? | 1. Husband only 2. A woman alone 3. Both 4. family |  |
| 113 | Is there any nearby health institution in your kebele? | 1. Yes 2. No 3. I do not know |  |
| 115 | What did you think about distance to health facilities? | 1. Long 2. Medium 3. Short 4. I do not know |  |
| 116 | How long does it take to go the nearest health institution? | 1. <30 min 2. >30 min. 3. I don’t know |  |
| 117 | What was your mode of transportation to health facilities? | 1. On foot 2. By motorcycle/bicycle/. 3. By car 4. Other |  |
| 118 | Had you heard about MCH service? | 1. Yes 2. No | If yes ans, next Qno.  114-115 |
| 119 | From where? | 1. By radio/TV 2. From health professional 3. School 4. Friends 5. Relatives 6. Others |  |
| 120 | If you heard by radio/TV, how often? | 1. Always 2. Once in a week 3. More than once a week |  |
| 121 | Have you heard of CBHI before? | Yes  No | If yes ans. Qn. 117 |
| 122 | Have you ensured in CBHI? | 1. Yes 2. No |  |

| **Socioeconomic: Wealth index measurement** | | | | | |
| --- | --- | --- | --- | --- | --- |
| 123 | Does your household have: |  | Response category | | Code |
|  |  | | Yes | No |  |
|  | 22.1. Electricity | | 1 | 2 |  |
|  | 22.2. Radio | | 1 | 2 |  |
|  | 22.3. Television | | 1 | 2 |  |
|  | 22.4. Non-mobile telephone | | 1 | 2 |  |
|  | 22.5. A table | | 1 | 2 |  |
|  | 22.6. A chair | | 1 | 2 |  |
|  | 22.7. Bed | | 1 | 2 |  |
| 124 | Does anyone of your household member own: | | Yes | No |  |
|  | 23.1. Does anyone of your household member own: Watch | | 1 | 2 |  |
|  | 23.2. Does anyone of your household member own: Mobile phone | | 1 | 2 |  |
|  | 23.3. Does anyone of your household member own: Bicycle | | 1 | 2 |  |
|  | 23.4. Does anyone of your household member own: Motorcycle? | | 1 | 2 |  |
|  | 23.5. Does anyone of your household member own: Bajaj | | 1 | 2 |  |
|  | 23.6. Does anyone of your household member own: An animal-drawn cart? | |  |  |  |
|  | 23.7. Does anyone of your household member own: A car/truck? | |  |  |  |
| 125 | Main material of the floor(observe) | | Yes | No |  |
|  | 24.1 Cemented | |  |  |  |
|  | 24.2. Others(Mud/crow dung, Earth/Sand, Wood, Ceramic) | |  |  |  |
| 126 | What is the main source of drinking water for your household? | | Yes | | No |
|  | 25.1. Piped water | |  | |  |
|  | 25.2. Others (Dug well, Water from spring, Rainwater, Tube well or borehole, Lake /pond/stream/canal) | |  | |  |
| 127 | What type of fuel does your household mainly use for Cooking? | | Yes | | No |
|  | 26.1. Electricity | |  | |  |
|  | 26.2. Natural gas | |  | |  |
|  | 26.3. Biogas | |  | |  |
|  | 26.4. Kerosene | |  | |  |
|  | 26.5. Charcoal | |  | |  |
|  | 26.6. Wood | |  | |  |
|  | 26.7. Straw /shrubs/grass | |  | |  |
|  | 26.8. Agricultural crop | |  | |  |
|  | 26.9. Animal dung | |  | |  |
| 128 | Does any member of this household have a bank or microfinance saving account | | | | |
| 129 | Does your household own? | | Yes | | No |
|  | 28.1. Does your household own milk Cows? | |  | |  |
|  | 28.2. Does your household own oxen? | |  | |  |
|  | 28.3. Does your household own hen? | |  | |  |
|  | 28.4. Does your household own Goat or Sheep? | |  | |  |
|  | 28.5. Donkey or Horse? | |  | |  |
|  | 28.6. Does your household own Mule? | |  | |  |
| 130 | Does this household own any agricultural land? | | Yes | | No |
| 131 | How many hectares of agricultural land? | | Yes | | No |
|  | Less than one hectar  Greater than one hectar | |  | |  |

| **S.n** | **Part two: Obstetric history related questions** | **Response** | | | | | | | **Skip** |
| --- | --- | --- | --- | --- | --- | --- | --- | --- | --- |
| 201 | Had you heard about family planning? | 1. Yes 2. No | | | | | | | If yes ans. Qn202 |
| 202 | Have you ever used any modern F/P method to delay or avoid getting pregnant of the last baby? | 1. Yes 2. No | | | | | | |  |
| 203 | How old were you when you got pregnant for the first time? | Completed age------------1.  I don’t know---99 | | | | | | |  |
| 204 | How many pregnancies did you have? | ---------------------------------- | | | | | | |  |
| 25 | Did the current pregnancy, wanted, mistimed or unwanted? | 1. Wanted 2. Unwanted 3. Mistimed | | | | | | |  |
| 206 | Have you ever attended ANC follow up for your current child? | 1. Yes 2. No | | | | | | |  |
| 207 | How many times in total did you receive antenatal care during this pregnancy? | 1. ______times 2. I don’t know | | | | | | |  |
| 208 | How many months pregnant were you when you first accessed antenatal care for that pregnancy? | ------------------------  I don’t know-----99 | | | | | | |  |
| 209 | Where did you get ANC service? | 1. Government Hospital 2. Health center 3. Health post 4. Private clinic 5. Home 6. Others(Specify)___________ | | | | | | |  |
| 210 | Whom did you see in your last ANC visit? Anyone else | 1. Doctor 2. Nurse 3. Midwife 4. Health officer 5. Health extension worker   99. I don’t know  Other(Specify)_______________ | | | | | | |  |
| 211 | Did you remember about the services provided during ANC? | Yes-----------1. No ---------------2 | | | | | | | If no skip to 213 |
| 212 | If yes, was the recommended ANC service provided during follow-up? | | | | | | | |  |
|  | (More than one ans. Possible) | Yes | | No | | | | |  |
|  | Blood pressure measured? |  | |  | | | | |  |
|  | Blood sample was taken? |  | |  | | | | |  |
|  | Urine sample was taken? |  | |  | | | | |  |
|  | TT2+ vaccination was provided? |  | |  | | | | |  |
|  | HIV test was provided? |  | |  | | | | |  |
|  | Health education was provided on (danger signs, nutrition, and birth preparedness)? |  | |  | | | | |  |
|  | Iron provision 90+ tablet? |  | |  | | | | |  |
| 213 | During any of antenatal visit were you told about birth preparedness plan? | **Yes** | | **No** | | | | |  |
|  |  |  | |  | | | | |  |
| 214 | Which plans were you told about?(More than one ans. Possible) |  | |  | | | | |  |
|  | Identified place of birth? |  | |  | | | | |  |
|  | Identified birth attendants? |  | |  | | | | |  |
|  | Identified the location of closest health facilities for birth? |  | |  | | | | |  |
|  | Prepared emergency transportation? |  | |  | | | | |  |
|  | Saved money for emergency? |  | |  | | | | |  |
|  | Identified labor and birth companion? |  | |  | | | | |  |
|  | Identified potential blood donor? |  | |  | | | | |  |
|  | Identified support person to look after the home and other children while women away? |  | |  | | | | |  |
| 214 | During (any of) your antenatal care visit(s), were you told about the signs of pregnancy complications or danger sign of pregnancy? | 1. Yes. 2. No | | | | | | | if yes ans. 215 |
| 215 | Which signs of pregnancy complications were you told about?(multiple response are possible) |  | Yes | | | | No | |  |
|  |  | Vaginal bleeding Vaginal gush of fluid  Severe headache  Blurred vision  Fever  Abdominal pain  Convulsion  Other(Specify) |  | | | |  | |  |
| 216 | When did you give birth of your last baby | ________/____/____ |  | | | |  | |  |
| 217 | Where did you deliver (name of Baby)? | 1. Government Hospital 2. Government Health Center 3. Health Post 4. Private Hospital/Clinic 5. Home   99. Other(Specify:________) | | | | | | |  |
| 218 | Who decided where you give birth and by whom? | 1. Myself 2. My husband 3. Both of us 4. Health Development Army 5. My relatives 6. Other(Specify)___________ | | | | | | |  |
| 219 | Which mode of transport did you mainly use to reach at the facility? | 1. Onfoot 2. Ambulance 3. Public transport 4. Cart 5. Traditional stretcher “*kareza”* 6. Other (specify)……………..…. | | | | | | |  |
| 220 | Did you go to the facility because there was a problem while you were in labor? or did you plan to deliver your baby in this facility? | 1. Yes, because there it was planned 2. No, because of problem during labour 3. 99.Don’t kno | | | | | | |  |
| 221 | What services provided during childbirth? (more than one answer possible)? | | | | | | | |  |
|  | **Newborn services** | | | | **Yes** | | | **No** |  |
|  | 1. Exercised skin to skin contact 2. Cord care 3. Initiated breast feeding with in one hours Immunization 4. Weight measurement 5. Others ------------ 6. No service at all | | | |  | | |  |  |
|  |  |  |  |  |  | | |  |  |
|  |  |  |  |  |  | | |  |  |
|  |  |  |  |  |  | | |  |  |
|  |  |  |  |  |  | | |  |  |
|  | **Maternal services** | | | | **Yes** | | | **No** |  |
|  | 1. Counseling on postpartum complication 2. Counseling on Postpartum 3. FP Provision of postpartum family planning 4. Others ----------------- 5. No service at all | | | |  | | |  |  |
|  |  |  |  |  |  | | |  |  |
|  |  |  |  |  |  | | |  |  |
|  |  |  |  |  |  | | |  |  |
|  |  |  |  |  |  | | |  |  |
| 222 | Did you receive professional assistance while you gave your last birth? | Yes ------1, No----------2 | | | | | | |  |
| 223 | What was the mode of delivery of this child? | 1. Spontaneous vaginal delivery 2. Instrumental/Cesarean delivery | | | | | | |  |
| 224 | If delivered through C/S, did you have your cesarean operation plan before you went into labor or decided after your labor had already started? | 1. Before labor started 2. After labor started 3. 99.Don’t know/can’t remember | | | | | | |  |
| 225 | Who attended the delivery? | 1. Doctor/nurse/midwife/health officer 2. Community HEW 3. Non health professional 4. Traditional birth attendants 5. I don’t know | | | | | | |  |
| 226 | What was the birth order of the last child? | ---------------------- | | | | | | |  |
| 227 | Did you have any information on danger signs of pregnancy? | 1. Yes 2. No | | | | | | |  |
| 228 | If your answer is yes, what are they?  (multiple response are possible)   1. Severe Vaginal bleeding 2. High grade fever 3. Foul smelling vaginal discharge 4. Urinary incontinence 5. Breast pain |  | | | | | | |  |
| 229 | Did you have any postnatal checkup in your last pregnancy? | 1. Yes  2. No | | | | | | | If no skip to Q.234 |
| 230 | How long after the delivery did the FIRST health check take place? | 1. Within 48 hours 2. With in 3-7 days 3. 7-2 weeks 4. 2-6 wks 5. I do not know | | | | | | |  |
| 231 | How many times you received the service? | ________________Times  99. Donot know | | | | | | |  |
| 232 | Where did you get the service? | 1. At own home 2. Health post 3. Health center 4. Public hospital 5. Private clinic/hospital 6. Other(Specify)…………… | | | | | | |  |
| 233 | What was your reason for attending health facilities for postnatal care? | 1. I was sick 2. Baby was sick 3. To check my health and the baby’s health 4. during child immunization 5. To get F/ planning 6. others(specify)_________ 7. No(0) Yes(1) | | | | | | |  |
| 234 | What type of professional personnel provides the postnatal care service? | 1. Doctor 2. Health Officers 3. Nurse 4. Health Extension Worker 5. Health professional but I don’t know her/his title. 6. I don’t know | | | | | | |  |
| 235 | What services was given to you and your newborn? (more than one answer possible) | **Newborn service** | | | | **yes** | | **No** |  |
|  |  | Cord care | | | |  | |  |  |
|  |  | Received necessary immunization | | | |  | |  |  |
|  |  | Weight measurement | | | |  | |  |  |
|  |  | Counseling on child feeding | | | |  | |  |  |
|  |  | Mention other | | | |  | |  |  |
|  |  | No service at all | | | |  | |  |  |
|  |  | **Maternal service** | | | | **yes** | | **No** |  |
|  |  | Counseling on family planning | | | |  | |  |  |
|  |  | Provision of family planning | | | |  | |  |  |
|  |  | Counseling on postpartum complication | | | |  | |  |  |
|  |  | Received anemia treatment | | | |  | |  |  |
|  |  | Mention others---------------- | | | |  | |  |  |
