# Supplementary material for: Levels and associated factors of the maternal healthcare continuum in Hadiya zone, Southern Ethiopia: A multilevel analysis
Source: PLoS One. 2022 Oct 10;17(10):e0275752. doi: 10.1371/journal.pone.0275752 (PMC9550044; doi:10.1371/journal.pone.0275752)
Supplement: Supplementary file 2 [file pone.0275752.s003.docx]

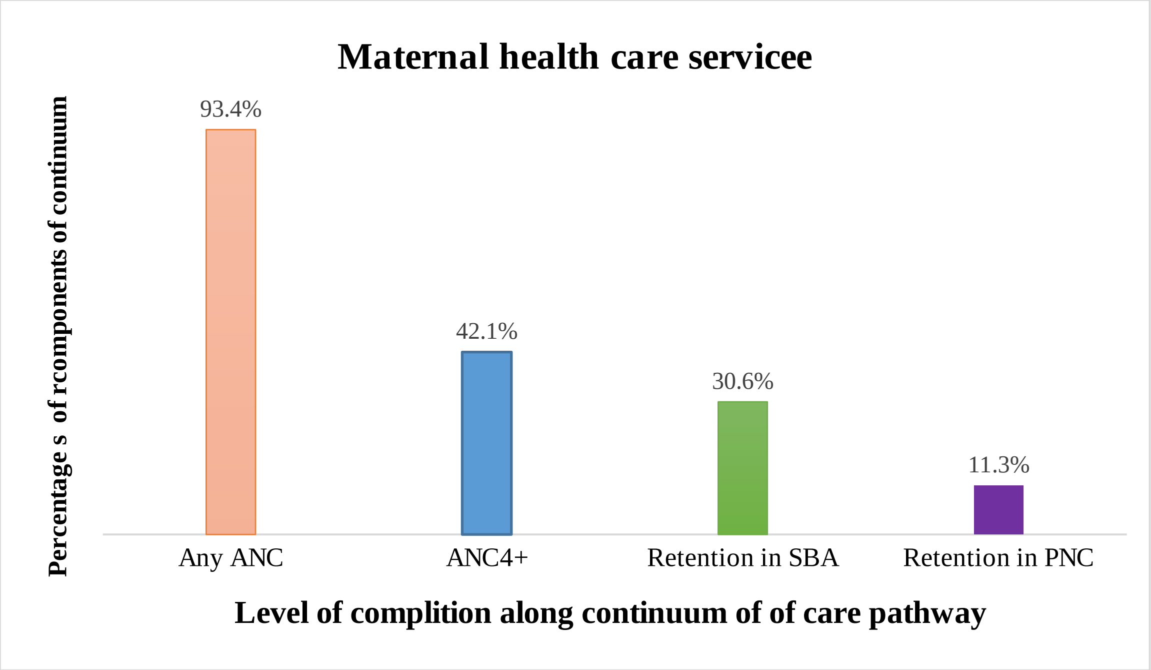
Maternal health care servicee
Percentage s of rcomponents of continuum
93.4%
42.1%
30.6%
11.3%
Any ANC
ANC4+
Retention in SBA
Retention in PNC
Level of complition along continuum of of care pathway
